# Supplementary material for: Carbon Nanotube Fibers Decorated with MnO2 for Wire-Shaped Supercapacitor
Source: Molecules. 2021 Jun 7;26(11):3479. doi: 10.3390/molecules26113479 (PMC8201185; doi:10.3390/molecules26113479)
Supplement: Supplementary file 1 [file molecules-26-03479-s001.zip › molecules-1225689-supplementary.pdf]

Supplementary information

Carbon nanotube fibers decorated with MnO_2 for wire-shaped supercapacitor

Luman Zhang [1], Xuan Zhang [1,*], Jian Wang [2], David Seveno [1], Jan Fransaer [1], Jean-Pierre Locquet [3] and Jin Won Seo [1,*]

1 Department of Materials Engineering, KU Leuven, Kasteelpark Arenberg 44 – bus 2450, B-3001 Leuven, Belgium
E-mail: lumanzhang1990@gmail.com. E-mail: xuan.zhang@kuleuven.be. E-mail: david.seveno@kuleuven.be. E-mail: jan.fransaer@kuleuven.be. E-mail: maria.seo@kuleuven.be.
2 Industrial Research Institute of Nonwovens & Technical Textile, College of Textiles & Clothing, Qingdao 266071, China
E-mail: wj8958@163.com
3 Department of Physics and Astronomy, KU Leuven, Celestijnenlaan 200D, B-3001 Leuven, Belgium
E-mail: jeanpierre.locquet@kuleuven.be

* Author to whom correspondence should be addressed.

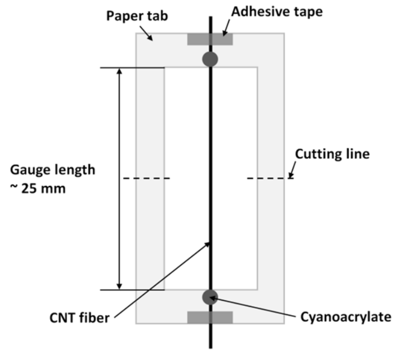

Figure S1. Specimen for single fiber tensile test.

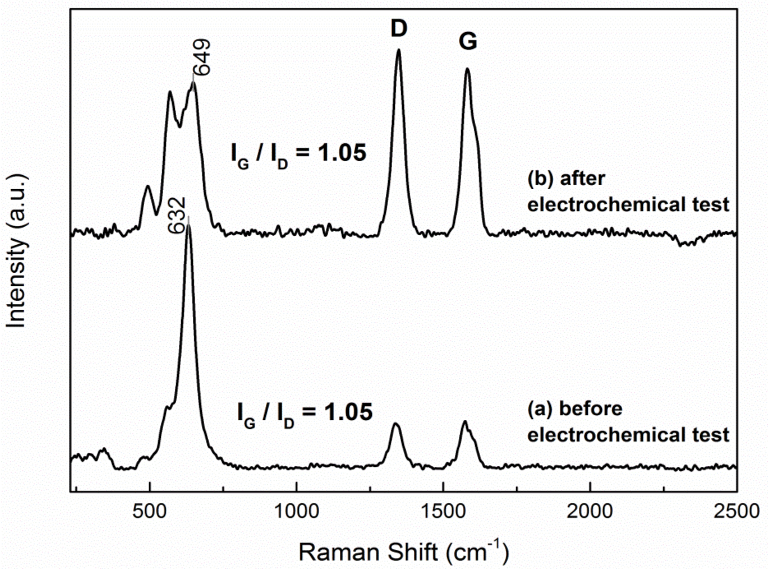

Figure S2. Raman spectra of CNT-MnO_2 fiber (10, 8) (a) before and (b) after electrochemical tests.

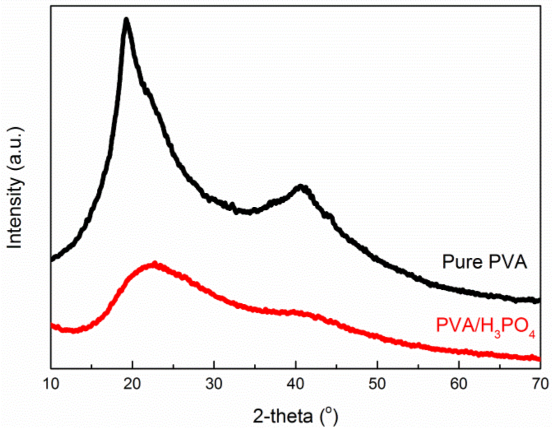

Figure S3. XRD analysis of the solid polymer electrolyte based on PVA.

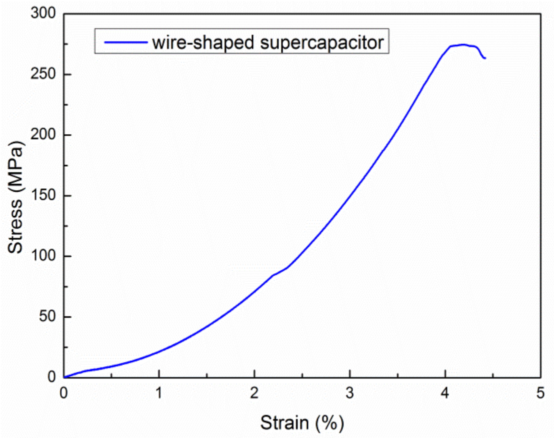

Figure S4. Strength-strain curve of the tested wire-shaped supercapacitor.

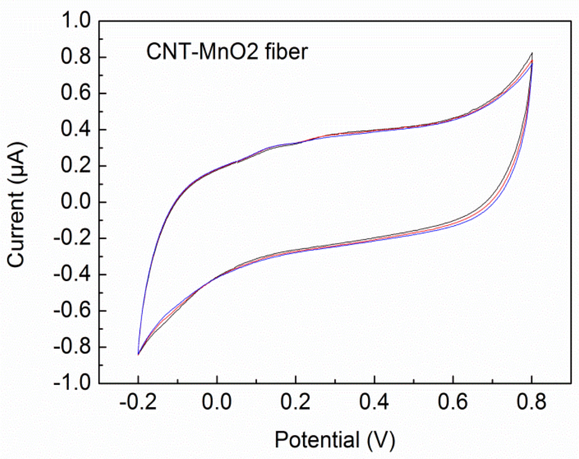

Figure S5. A non-quasi-rectangular CV curve of a CNT-MnO_2 fiber electrode, operating voltage window: -0.2 – 0.8V.
